# Breast tumour cell-induced down-regulation of type I collagen mRNA in fibroblasts

**DOI:** 10.1038/sj.bjc.6690821

**Published:** 1999-12

**Authors:** G Fenhalls, M Geyp, D M Dent, M I Parker

**Affiliations:** Departments of Medical Biochemistry, Surgery, Faculty of Health Sciences, University of Cape Town, Observatory, 7925, Republic of South Africa

**Keywords:** breast cancer, extracellular matrix, cell–cell interaction, collagen gene expression

## Abstract

This study investigated the modulation of type I collagen gene expression in normal fibroblasts by breast tumour cells. Northern analysis of total RNA extracted from stages I, II and III breast tumour tissue revealed that collagen mRNA levels were elevated in stage I tumours compared to the adjacent normal breast tissues, whereas they were decreased in stages II and III breast tumours. This aberrant collagen gene expression was confirmed by non-radioactive RNA:RNA in situ hybridization analysis of 30 breast carcinomas which localized the production of type I collagen mRNA to the stromal fibroblasts within the vicinity of the tumour cells. In order to determine whether the tumour cells were directly responsible for this altered collagen production by the adjacent fibroblasts, breast tumour cell lines were co-cultured with normal fibroblasts for in vitro assessment of collagen and steady-state collagen RNA levels. Co-culture of tumour cells and normal fibroblasts in the same dish resulted in down-regulation of collagen mRNA and protein. Treatment of the fibroblasts with tumour-cell conditioned medium also resulted in decreased collagen protein levels but the mRNA levels, however, remained unaltered. These results suggested that the tumour cells either secrete a labile ‘factor’, or express a cell surface protein requiring direct contact with the fibroblasts, resulting in down-regulation of collagen gene expression. Modulation of the ECM is a common characteristic of invading tumour cells and usually involves increased production of collagenases by the tumour cells or stromal fibroblasts. This study showed that tumour cells were also able to modulate collagen mRNA production by stromal fibroblasts, which may facilitate tumour cell invasion and metastasis. © 1999 Cancer Research Campaign


*British Journal of Cancer* (1999) **81**(7), 1142–1149
© 1999 Cancer Research Campaign
Article no. bjoc.1999.0821

### Breast tumour cell-induced down-regulation of type I collagen mRNA in fibroblasts

**G Fenhalls[1], M Geyp[1], DM Dent[2] and MI Parker[1]**

Departments of [1]Medical Biochemistry and [2]Surgery, Faculty of Health Sciences, University of Cape Town, Observatory, 7925, Republic of South Africa

**Summary** This study investigated the modulation of type I collagen gene expression in normal fibroblasts by breast tumour cells. Northern analysis of total RNA extracted from stages I, II and III breast tumour tissue revealed that collagen mRNA levels were elevated in stage I tumours compared to the adjacent normal breast tissues, whereas they were decreased in stages II and III breast tumours. This aberrant collagen gene expression was confirmed by non-radioactive RNA:RNA in situ hybridization analysis of 30 breast carcinomas which localized the production of type I collagen mRNA to the stromal fibroblasts within the vicinity of the tumour cells. In order to determine whether the tumour cells were directly responsible for this altered collagen production by the adjacent fibroblasts, breast tumour cell lines were co-cultured with normal fibroblasts for in vitro assessment of collagen and steady-state collagen RNA levels. Co-culture of tumour cells and normal fibroblasts in the same dish resulted in down-regulation of collagen mRNA and protein. Treatment of the fibroblasts with tumour-cell conditioned medium also resulted in decreased collagen protein levels but the mRNA levels, however, remained unaltered. These results suggested that the tumour cells either secrete a labile ‘factor’, or express a cell surface protein requiring direct contact with the fibroblasts, resulting in down-regulation of collagen gene expression. Modulation of the ECM is a common characteristic of invading tumour cells and usually involves increased production of collagenases by the tumour cells or stromal fibroblasts. This study showed that tumour cells were also able to modulate collagen mRNA production by stromal fibroblasts, which may facilitate tumour cell invasion and metastasis.
© 1999 Cancer Research Campaign

**Keywords:** breast cancer; extracellular matrix; cell–cell interaction; collagen gene expression

One of the major aspects of tumour cell invasion and metastasis is the interaction between cancer cells and the surrounding extracellular matrix (ECM). This interaction involves all the components of the ECM, of which type I collagen is the most abundant and is synthesized predominantly by fibroblasts. Some studies have demonstrated that type I collagen may also be produced by epithelial cells (Al-Adanani et al, 1975; Roesel et al, 1978; Sakakibara et al, 1982; Liotta et al, 1983; Ohtani et al, 1992).

Collagen is involved in tumour progression in two very different ways. The desmoplastic response to a tumour results in excess deposition of collagen around the tumour. Conversely, collagen degradation and decreased synthesis allow invasion of tumour cells through the stroma. These processes may take place concurrently (van der Hooff, 1988). Desmoplasia, the increased deposition of stromal collagen, often results in the ‘hardening’ and ‘encapsulation’ of the tumour (Pucci-Minafra et al, 1986) and is thought to be a host reaction to tumour cell invasion (Ohtani et al, 1992; Hewitt et al, 1993). It has been shown to occur in cancers such as diffuse infiltrating gastric carcinomas and infiltrating (scirrhous) carcinomas of the breast (Ohtani et al, 1992). In the desmoplastic stroma of scirrhous breast carcinomas, collagen type I is the most abundant protein (Barsky et al, 1982). The cause of the desmoplasia is unknown and could be a response by the host in order to isolate the tumour and prevent it from further growth and possible invasion (Basset et al, 1990). Alternatively, the tumour cells might secrete factors that induce desmoplasia, thereby reducing access to the tumour by host lymphocytes, macrophages and other immune regulators (Barsky and Gopalakrishna, 1987).

Degradation of the ECM is dependent on specific interactions between tumour and host cells. Type I collagen, for example, is degraded by interstitial collagenase or matrix metalloprotease-1 (MMP-1), which is produced by a number of cell types such as tumour cells, fibroblasts, mast cells, leucocytes and macrophages (Pauli et al, 1983; Biswas, 1984). Tumour cells can indirectly alter the ECM by modulating fibroblast functions such as the secretion of an extracellular matrix metalloproteinase-inducer (EMMPRIN), which stimulates fibroblasts to produce collagenases (Biswas, 1982).

In this study, the relationship between type I collagen synthesis and breast cancer stage was investigated. Type I collagen gene expression was assessed in a number of normal and breast tumour tissues by Northern analysis and RNA:RNA in situ hybridization in order to determine whether there is a correlation between tumour stage and collagen gene expression. In situ hybridization analysis localized the $\alpha$1(I) and $\alpha$2(I) collagen mRNA to the stromal fibroblasts adjacent to the tumour cells and not the tumour cells in the breast carcinoma sections. Fibroblasts adjacent to stage I tumours exhibited increased collagen mRNA levels compared to the adjacent normal tissue, whereas in stage II and III tumours they showed decreased mRNA levels. Furthermore, in vitro co-culture of normal fibroblasts with breast tumour cell lines resulted in down-regulation of collagen mRNA synthesis and protein production by the fibroblasts. It would appear that the tumour cells and fibroblasts need to be in close proximity in order for the down-regulation of collagen mRNA to occur.

*Received 20 November 1998*
*Revised 20 April 1999*
*Accepted 22 April 1999*

*Correspondence to:* MI Parker

These results suggest that the tumour cells are able to regulate collagen production by the adjacent normal fibroblasts, either directly or indirectly, resulting in decreased ECM production, which would facilitate tumour cell invasion and subsequent metastasis.

### MATERIALS AND METHODS

### Breast tumour tissue

Breast tumour tissue samples were collected from mastectomy specimens in the theatre at Groote Schuur Hospital, Cape Town, Republic of South Africa. The tumours were all of primary infiltrating ductal type and samples were taken from the centre of the tumour. Adjacent normal breast tissue, excised at a distance from the tumour, was obtained from the same patient. The material was divided, one half was immediately placed in liquid nitrogen and stored at –70°C until further use, while the other half was embedded in paraffin for pathological analysis.

### Northern blot analysis

Breast tissue was homogenized on ice in guanidine isothiocyanate solution after which the RNA was isolated as described by Chomczynski and Sacchi (1987). Five micrograms of RNA were electrophoresed on a 1% agarose gel containing 8% formaldehyde, transferred to Hybond-N membranes which were then prehybridized for 4 h at 42°C in 10% dextran sulphate, 5 × standard saline citrate (SSC), 50 mM sodium pyrophosphate, 5 × Denhardts solution, 50% formamide, 0.1 mg ml$^{-1}$ herring sperm DNA and 0.1% sodium dodecyl sulphate (SDS).

α1(I) collagen cDNA (Chu et al, 1982), α2(I) collagen cDNA (Myers et al, 1981) and pGEM β-actin probes were labelled with $^{32}$P-dCTP by nick translation (Amersham). Approximately $1 \times 10^6$ cpm ml$^{-1}$ of denatured probe was added to the prehybridization solution and the membranes were incubated overnight at 42°C. The filters were washed twice for 15 min each at room temperature in 2 × SSC, 0.1% SDS followed by two washes for 15 min each at 65°C in 0.1 × SSC, 0.1% SDS. Membranes were exposed to X-ray film and quantitated by densitometry using the β-actin signal as a control. All membranes were boiled in 0.1% SDS to remove the hybridized probe for subsequent rehybridization to the second probe.

### RNA : RNA in situ hybridization

Paraffin-embedded primary infiltrating ductal carcinomas and their adjacent normal tissue were cut into 5-μm sections using a microtome and consecutive sections were applied to RNAase-free slides previously coated with 5 μg ml$^{-1}$ aminopropyltriethoxysilane. Sections were deparaffinized in xylene, rehydrated through graded ethanols and finally phosphate-buttered saline (PBS). The sections were treated with 1 μg ml$^{-1}$ proteinase K in 10 mM Tris–HCl (pH 7.5), 5 mM EDTA for 45 min at 37°C. After washing in PBS, the sections were refixed in 0.4% paraformaldehyde, acetylated in a 400:1 (v/v) solution of triethanolamine:acetic anhydride for 10 min, rinsed in PBS, dehydrated in graded ethanols and air-dried before hybridization. Sections were incubated in a prehybridization mixture containing 25% dextran sulphate, 25 mM Tris–HCl (pH 8.0), 2.5 × Denhardts solution, 2.5 mM EDTA, 25 mM dithiothreitol (DTT) 1.25 mg ml$^{-1}$ herring sperm DNA, 0.06 mg ml$^{-1}$ tRNA and 50% deionized formamide for 2 h at 50°C.

The hybridization probes were prepared as follows. A 160 base pair XbaI-SacI fragment from HF32 (α2(I) collagen) and the 581 base pair *Eco*RI-*Sal*I fragment from HF677 (α1(I) collagen) were subcloned into pGEM3 (Promega). The 500 base pair fragment from pGEMβ-actin was released by digestion with *Pvu*II and *Sal*I (Boehringer Mannheim). The appropriate fragments were excised from the plasmids and used as templates to transcribe digoxigenin-labelled antisense RNA using T7 RNA polymerase (Boehringer Mannheim). The vector sequences (pGEM3) were used as a negative control to ensure that the hybridization signals were specific.

In situ hybridization was performed as described by Hoefakker et al (1995). Briefly, the digoxigenin-labelled riboprobe (5 ng μl$^{-1}$) was added to a hybridization mixture containing 20% dextran sulphate, 12.5 mM Tris–HCl (pH 8.0), 2.5 × Denhardts solution, 2.5 mM EDTA, 2.5 mM DTT, 0.01 mg ml$^{-1}$ herring sperm DNA, 0.002 mg ml$^{-1}$ tRNA and 50% deionized formamide. The sections were hybridized for 16–18 h at 50°C in a humidified chamber, after which they were washed twice in 2 × SSC for 15 min each at room temperature, followed by two washes in 0.1 × SSC at 43°C for 15 min each. The sections were then incubated in 100 mM Tris–HCl (pH 7.5), 150 mM sodium chloride for 5 min, blocked with 2% normal sheep serum for 20 min at 37°C and washed in 0.05% Tween-20. Anti-digoxigenin antibody conjugated to alkaline phosphatase (Boehringer Mannheim) was incubated with the sections for 30 min at 37°C and the signal detected using NBT and X-Phosphate as described by the manufacturers (Boehringer Mannheim). Sections were dehydrated in ethanol, placed in xylene, covered with a coverslip and viewed under a light microscope.

### Histopathological data

Infiltrating ductal carcinomas were staged according to the TNM system where T is the tumour size, N is the number of lymph nodes involved and M is the presence of distant metastases (Harris et al, 1992). In this system, stage I tumours were < 2 cm, stage II tumours were > 2 cm but < 5 cm and stage III tumours were > 5 cm. Histological data were determined by microscopic examination of haematoxylin and eosin-stained sections and staged independently by two pathologists.

### Cell culture

Normal breast tissue was cut into very fine pieces and placed in 30-mm sterile Petri dishes under a cover slip. The breast fibroblasts (BRF) were cultured in minimal essential medium (MEM) containing 10% heat-inactivated fetal calf serum (FCS), 100 μg ml$^{-1}$ penicillin and 100 units ml$^{-1}$ streptomycin. WI-38 human lung fibroblasts, and the breast tumour epithelial cell lines MDA-MB- 231, MCF-7, T47D and ZR-75-1 were also cultured in the same medium.

### Co-culture of normal fibroblasts and tumour cell lines

A mixture of 20 000 fibroblasts (BRF or WI-38) and 20 000 tumour cells (MDA-MB-231, MCF-7, T47D or ZR-75-1) were seeded in 60-mm tissue culture dishes and incubated in MEM

© 1999 Cancer Research Campaign

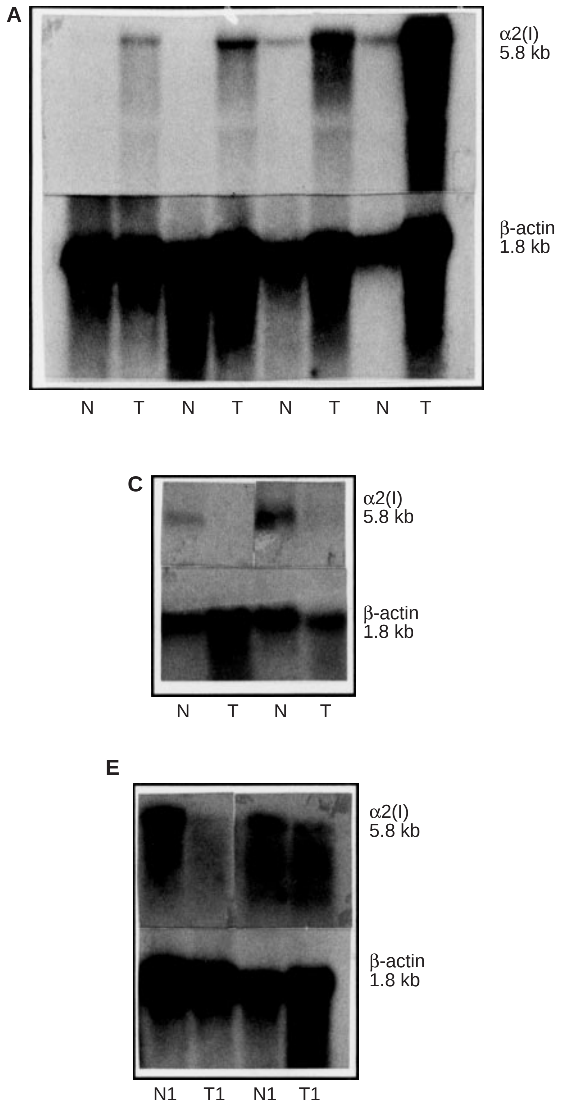

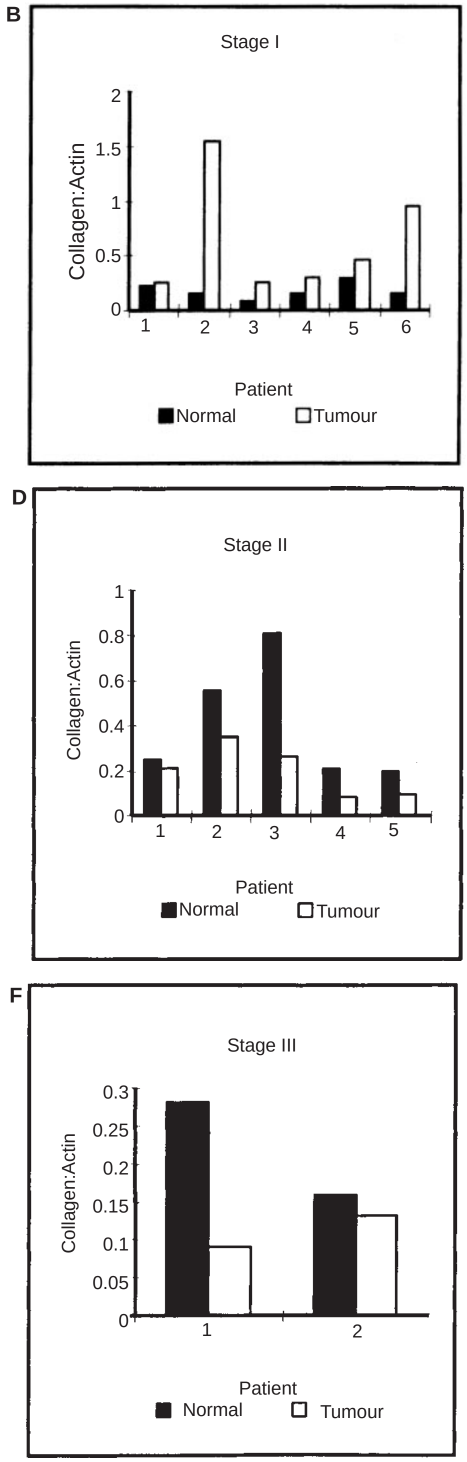

**Figure 1** Northern blot analysis of RNA extracted from stages I, II and III breast tumours and from the adjacent normal tissue. Total RNA was extracted from breast tumours (T) and the adjacent normal (N) tissue of each patient and hybridized with the nick-translated Hf32, full length α2(I) collagen cDNA probe as described in Materials and Methods. The membranes were stripped and re-hybridized with a nick translated β-actin cDNA probe, washed and exposed to X-ray film for 24 h. **A**, **C** and **E** are autoradiographs from representative Northern blots of total RNA from stage I, II and III tumours respectively. Note that not all of the patient samples are shown. A summary of the expression of α2(I) collagen mRNA relative to β actin mRNA from all the patients with stages I, II and III tumours are shown in **B**, **D** and **F** respectively containing 10% FCS and the above antibiotics for 48 h prior to harvesting for determination of collagen protein and mRNA levels.

### Collagen synthesis in fibroblasts cultured in tumour-cell conditioned medium

Conditioned media were prepared from MDA-MB-231, T47D, MCF-7 and ZR-75-1 breast cancer cell lines as described by Biswas (1982). After 48 h of incubation the medium was removed, the cell layers rinsed twice with PBS and serum-free MEM was added. After 48 h the serum-free medium was removed and centrifuged for 1 h to remove any cellular debris. The medium was dialysed overnight at 4°C against distilled water, lyophilized, reconstituted in one-tenth the original volume of sterile water and sterilized by filtration.

Breast fibroblasts were plated at a density of 40 000 cells per well in 24-well dishes and grown to 80% confluency. The cells

© 1999 Cancer Research Campaign were incubated in 1X conditioned medium at 37°C for 32 h and for a further 16 h in the presence of 50 μg $ml^{-1}$ ascorbate, 50 μg $ml^{-1}$ β-aminopropionitrile and 10 μCi $ml^{-1}$ $^3$H-proline in order to label the proteins prior to harvesting. Collagen was harvested from the medium and quantitated using the collagenase assay as described by Peterkofsky and Diegelmann (1974). The means and standard deviations of three different experiments were calculated.

### RESULTS

### Northern blot analyses

Total RNA was extracted from 30 primary infiltrating ductal carcinomas and their adjacent normal tissues and probed for α1(I) and α2(I) collagen mRNA by Northern blot analysis. The expression of α2(I) collagen and β-actin mRNAs for several of the patients are shown in Figure 1. Stage I breast carcinomas had increased levels α2(I) collagen mRNA when compared to the adjacent normal tissue (Figure 1 A,B). Stages II and III breast tumours, however, had decreased α2(I) collagen mRNA levels when compared to those in adjacent normal tissue (Figure 1 C–F). Levels of α1(I) collagen mRNA were similar to those of the α2(I) collagen mRNA (data not shown). All the results are expressed relative to β-actin which was used to correct for any fluctuations in RNA loading as well as to control for RNA degradation.

### In situ hybridization analyses

In order to confirm the changes in collagen steady-state mRNA levels detected by Northern blot analysis and to localize the cells in which these changes occurred, breast tumour sections from each of stages I, II and III and the corresponding adjacent normal tissue were subjected to non-radioactive in situ hybridization (Figure 2). Sections of a stage I tumour and the adjacent normal tissue were hybridized with an α2(I) collagen riboprobe (Figure 2 A,B). The normal tissue showed no histological abnormalities and was characterized by intact mammary ducts (md), whereas the tumour tissue contained fragmented collagen fibrils in the stroma and numerous multinucleated tumour cells. The fibroblasts (indicated by arrows) present in the stroma surrounding the tumour cells (t), as well as those in the adjacent normal tissue, were positive for α2(I) collagen mRNA, while the tumour cells themselves were negative. Both the stromal fibroblasts and epithelial cells were positive for β-actin in the normal and tumour tissue sections (data not shown). These results demonstrated that the staining for collagen mRNA was specific to the fibroblasts in the stroma and not to the epithelial cells.

Sections of stage II tumour and normal tissue clearly showed fibroblasts (arrows) lodged between the collagen fibrils (c) (Figure 2 C,D). A large number of fibroblasts were also located close to the tumour cells. The fibroblasts present in the normal tissue adjacent to the tumour were positive for α2(I) collagen mRNA, while those fibroblasts in the tumour were negative (indicated by an arrow). The fibroblasts and tumour cells that were negative for collagen mRNA were positive for β-actin mRNA (data not shown). This indicated that those fibroblasts in the tumour tissue were producing β-actin but not collagen mRNA.

Figure 2E and 2F show sections of a stage III tumour and the adjacent normal tissue respectively. The stroma in the normal tissue section was fairly intact, consisting of intact collagen fibrils (c) and fibroblasts (arrows), while the tumour stroma (t) contained infiltrating tumour cells and lymphocytes as well as fibroblasts. As was shown for the stage II tumour above, the fibroblasts present in the adjacent normal tissue were positive for α2(I) collagen mRNA, while those in the tumour tissue were negative. Both the fibroblasts and tumour cells were positive for β-actin mRNA (data not shown).

**Table 1.** Thirty tissue sections from stages I, II and III breast carcinomas were analysed for collagen and β-actin gene expression by RNA:RNA in situ hybridization

| Tumour stage | No. of patients | Collagen gene expression | β-actin gene expression |
|---|---|---|---|
| I | 10 | +++ | +++ |
| II | 10 | – | +++ |
| III | 10 | – | +++ |

Level of expression in fibroblasts in the tumour section was determined visually and scored as either weaker (–) or stronger (+++) than fibroblasts in the normal tissue sections.

A summary of results for 30 infiltrating breast carcinomas is shown in Table 1. All ten stage I carcinomas exhibited increased α1(I) and α2(I) collagen mRNA levels when compared to the normal tissues, while stages II and III carcinomas had decreased collagen gene expression. All tissue sections were probed with pGEM3 riboprobe as a negative control and no signal was detected indicating no non-specific binding of the riboprobes (data not shown). Consecutive sections were also probed with an α1(I) collagen riboprobe and similar results to those for α2(I) collagen mRNA were obtained (data not shown).

### In vitro co-culture of fibroblasts with breast tumour cell lines

In order to determine whether the tumour cells affected collagen gene expression in normal fibroblasts, various breast tumour cell lines were co-cultured with normal human breast fibroblasts (BRFs). The exact stage of these tumour cell lines are unknown but they are probably derived from stage IV tumours (ATCC). BRFs co-cultured with the breast tumour cell lines MDA-MB-231, MCF-7, T47D and ZR-75-1 exhibited a greater than 50% decrease ($P < 0.05$) in collagen protein as assessed by the collagenase assay (Figure 3A). Since the breast tumour cells do not produce collagen (data not shown) any contribution by these cells can be ignored. The breast tumour cell lines were also co-cultured with WI-38 lung fibroblasts and a similar down-regulation of collagen synthesis was observed (Figure 3B).

Total α2(I) collagen mRNA in co-cultures of WI-38 fibroblasts and breast tumour cells was shown to be decreased ($P < 0.05$) as assessed by Northern blot analysis (Figure 4). All samples were corrected for cell number. These results indicated that the tumour cells were responsible for modulating collagen gene expression in the fibroblasts.

### Culture of breast fibroblasts with conditioned medium from breast tumour cell lines

In order to determine whether tumour cells secreted a stable soluble factor(s) which was responsible for the down-regulation of type I collagen gene expression, BRFs were incubated for 48 h with conditioned medium prepared from the breast tumour cell

© 1999 Cancer Research Campaign

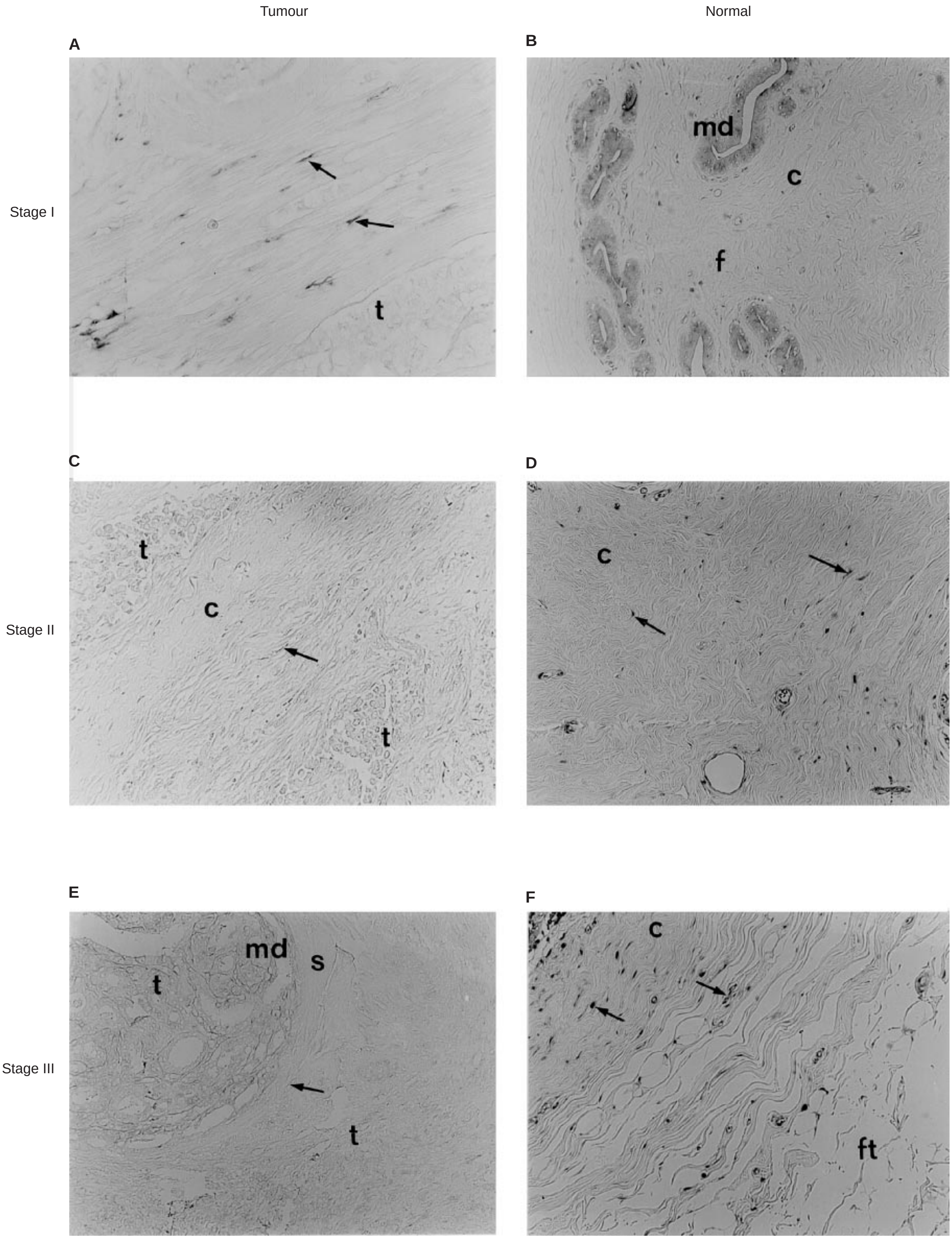

**Figure 2** RNA:RNA in situ hybridization analysis of α2(I) collagen mRNA in stages I, II and III breast tumours and their adjacent normal tissues. Consecutive sections of breast tumour explants were cut from a wax block and hybridized with the α2(I) collagen probe (**A–F**) or stained with haemotoxylin and eosin in order to study the morphology (data not shown). The stage I tumour section (**A**) clearly shows the tumour cells (t) that have infiltrated the stroma containing several stromal fibroblasts (arrows). The normal tissue (**B**) is characterized by normal mammary ducts (md) as well as intact collagen fibrils (c) with numerous stromal fibroblasts (f). The stromal fibroblasts (arrows) in the tumour (**A**) were positive for α2(I) collagen mRNA (blue-purple staining of digoxigenin-labelling) while the tumour cells (t) were not. Sections of a stage II breast carcinoma and the adjacent normal tissue are shown in **C** and **D** respectively. The tumour section (**C**) contains tumour cells (t), which have infiltrated the stroma (c) and the fibroblasts (arrow) are negative for α2(I) collagen mRNA. The normal tissue (**D**) shows intact collagen fibres (c) interspersed with fibroblasts staining positive for α2(I) collagen mRNA (arrows). Sections from a stage III breast tumour and adjacent normal tissue are shown in **E** and **F**. The tumour tissue (**E**) consists mostly of tumour cells (t) and a region of intact stroma (s), containing a few fibroblasts (arrow), next to the mammary duct (md) which is filled with tumour cells (t). The normal tissue shows intact collagen fibrils (c) with several fibroblasts (arrow) as well as fragmented tissue (ft). Fibroblasts (arrow) in the normal tissue (**F**) stained positive for α2(I) collagen mRNA whereas the fibroblasts at the periphery of the mammary duct in (**E**) were negative. Tumour cells were also negative. Total magnification for each section was 100×

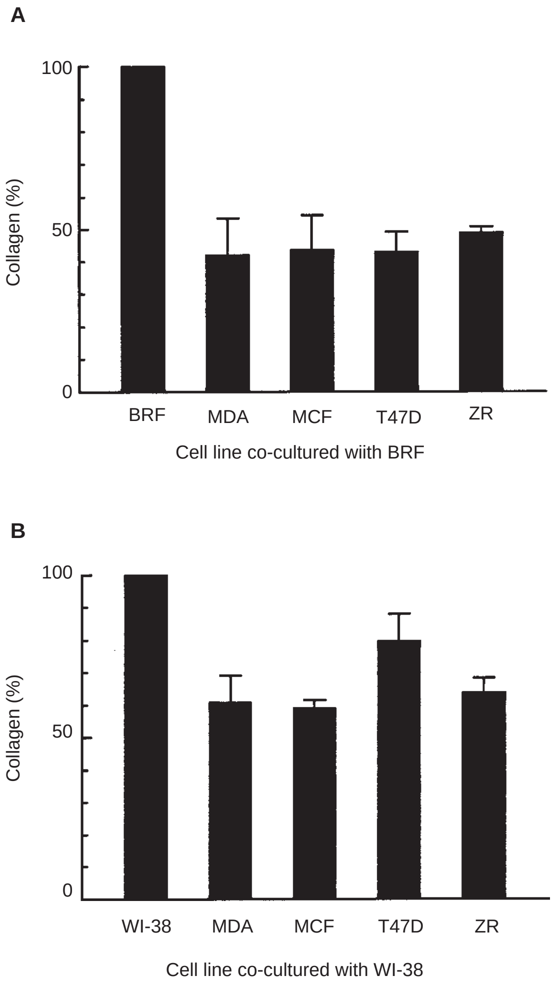

**Figure 3** Synthesis of collagen in (**A**) breast fibroblasts (BRF) or (**B**) WI-38 lung fibroblasts co-cultured with breast tumour cell lines. Twenty thousand BRF or WI-38 fibroblasts and 20 000 of the indicated breast tumour cell lines MDA-MB-231 (MDA), MCF-7 (MCF), T47D and ZR-75-1 (ZR) were plated onto 60 mm dishes and cultured for 32 h at which time 10 μCi $ml^{-1}$ $^{3}H$-proline, 50 μg $ml^{-1}$ ascorbate a 50 μg $ml^{-1}$ β-aminoproprionitrile was added in order to label the collagen synthesized and the cells were further incubated for 16 h. Collagen in the culture medium was quantitated using the collagenase assay as described in Materials and Methods. Relative collagen production in co-cultures was determined as a percentage of the collagen produced by BRF or WI-38 cells cultured alone. Bars: standard error of the mean of three separate experiments. Statistical significance was determined using an unpaired Student's *t*-test lines and the levels of collagen protein and mRNA were determined.

Incubation of BRFs with tumour cell conditioned medium resulted in decreased ($P < 0.05$) collagen protein levels (Figure 5A), whereas α2(I) collagen mRNA levels did not change significantly (Figure 5B). Collagenase activity was found to be present in the tumour cell conditioned media after co-culture with the fibroblasts (data not shown) which accounted for the discrepancy between collagen protein and mRNA levels. Similar results were found in studies by Biswas (1984).

These results suggested that, while collagenases induced by the tumour cells were contributing to the decreased collagen protein levels observed in fibroblasts cultured in tumour cell conditioned media, the conditioned media alone could not induce down-regulation of collagen mRNA observed in the co-culture of fibroblasts with tumour cells.

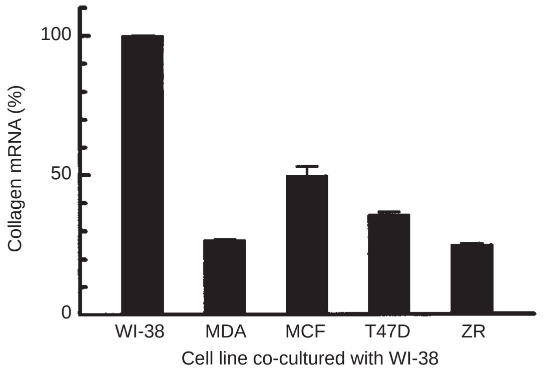

**Figure 4** Synthesis of collagen mRNA in WI-38 lung fibroblasts co-cultured with tumour cells. Equal numbers of WI-38 fibroblasts and either MDA-MB-231 (MDA), MCF-7 (MCF), T47D or ZR-75-1 (ZR) cells were co-cultured in 100 mm dishes for 48 h. Total RNA was isolated from co-cultures and Northern blot analysis was carried out as described in Materials and Methods. Relative collagen mRNA levels in co-cultures (after correction for contributing cell number) was determined as a percentage of the collagen mRNA in WI-38 cells cultured alone. Bars: standard error of the mean of three separate experiments. Statistical significance was determined using an unpaired Student's *t*-test

### DISCUSSION

The Northern blot and in situ hybridization data on stages I, II and III infiltrating ductal breast carcinomas indicated that tumour cells can modulate type I collagen production. The elevation of type I collagen mRNA levels in stage I breast tumours is most likely due to the desmoplastic response (Noel et al, 1992). Breast tumours which are associated with desmoplasia are sometimes known as ‘scirrhous carcinomas’ (Noel et al, 1992). The cause of the desmoplastic response remains unclear. One possible explanation for the results presented in this study is that the excess collagen production is a host defence in response to tumour cell invasion rather than a tumour cell response for survival and growth.

There has been considerable controversy as to whether the tumour cells themselves (Al-Adanani et al, 1975; Niitsu et al, 1988), or the fibroblasts in the vicinity of the tumour cells, are stimulated to produce type I collagen (Barsky et al, 1982; Ohtani et al, 1992; Hewitt et al, 1993). Early studies claimed that the excess production of type I collagen is not due to increased collagen synthesis by the stromal fibroblasts, but that it is produced by the breast cancer cells themselves, which suggested that this response was an inappropriate rather than a deliberate host response (Al-Adanani et al, 1975). Subsequent studies have shown that stromal fibroblasts do, in fact, produce the excess collagen and that the epithelial cells are not responsible for the desmoplastic effect (Barsky et al, 1982; Ohtani et al, 1992). The in situ hybridization results presented in this study clearly demonstrated that the host fibroblasts within the vicinity of the tumour were responsible for the increased production of type I collagen mRNA in the stage I breast carcinomas.

Nakanishi and co-workers (Nakanishi et al, 1994), using clones of mouse Lewis lung carcinoma-derived cell lines with different metastatic potentials, found an inverse relationship between the host stromal response and spontaneous lung metastasis. These results are similar to those reported in the present study where collagen levels in stages I, and II/III are similar to those in the low and high metastatic cells respectively. Our study found that the fibroblasts were still present at the advanced stages of breast

*© 1999 Cancer Research Campaign*

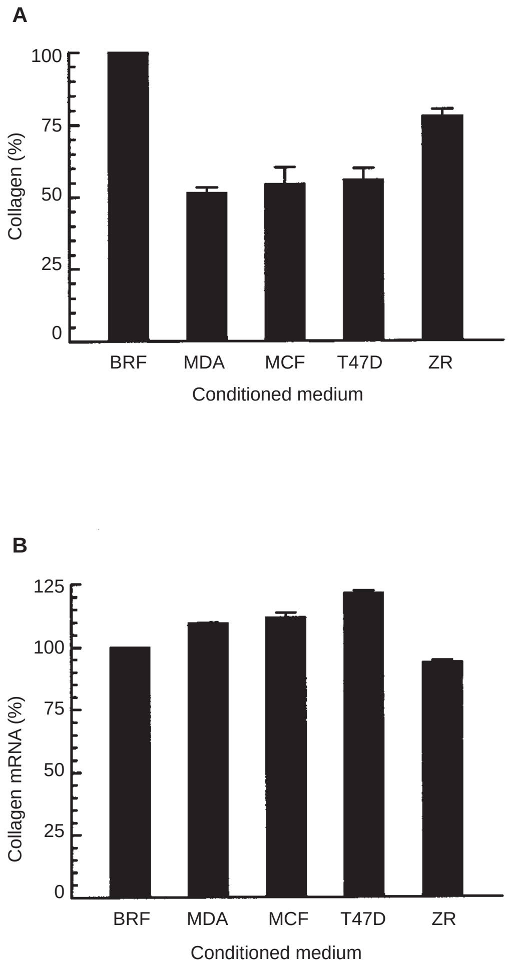

**Figure 5** The effect of tumour cell conditioned medium on collagen synthesis. Collagen synthesis by breast fibroblasts (BRF) incubated with conditioned media from either BRF, MDA-MB-231 (MDA), MCF-7 (MCF), T47D or ZR-75-1 (ZR) cells is shown in (**A**). Collagen protein levels in the culture media were determined using the collagenase assay described in Materials and Methods. (**B**) shows relative levels of α2(I) collagen mRNA in breast fibroblasts incubated with conditioned media from MDA-MB-231 (MDA), MCF7 (MCF) and ZR-75-1 (ZR) tumour cell lines. Alpha 2(I) collagen RNA levels were determined by Northern blot analysis. Relative expression was determined as a percentage of the collagen protein or mRNA expressed by BRFs incubated with fibroblast conditioned medium. Bars: standard error of the mean of three separate experiments. Statistical significance was determined using an unpaired Student's *t*-test cancer, but that they were not producing collagen mRNA. While Kauppila et al (1998) studied the expression of collagen in different grades of breast tumour and found increased collagen expression with increased de-differentiation, our study focused on the stage of tumour which takes into account the biological quality of tumour progression, growth and metastasis. The data presented in this study suggest that desmoplasia is associated with the early stages of the disease (stage I) and that collagen mRNA is down-regulated in the later stages (II and III), which possibly facilitates tumour cell invasion and eventual metastasis. This is in agreement with the study by Hewitt and co-workers (Hewitt et al, 1993), who reported the absence of type I collagen mRNA at the invasive edge of colorectal tumours.

The in situ hybridization data suggested that the tumour cells may be responsible for the observed down-regulation of collagen mRNA in the adjacent fibroblasts since the fibroblasts in the direct vicinity of the tumour cells exhibited decreased levels of collagen mRNA. The other fibroblasts (as shown in the adjacent normal sections of stage II and III tumours) were unaffected and continued producing collagen mRNA. In vitro studies involving co-culture of normal fibroblasts with several established breast tumour cell lines supported this hypothesis. Co-culture of breast or lung fibroblasts with tumour cells (which themselves do not produce collagen) resulted in down-regulation of collagen protein and mRNA production by the fibroblasts. These results indicated that the tumour cells were down-regulating collagen production by the adjacent fibroblasts.

Conditioned medium experiments suggest that the down-regulation of collagen mRNA was not due to a soluble factor secreted by the tumour cells, although it is possible that a factor is secreted in very low concentrations by the tumour cells such that it may be diluted in the conditioned media. Thus, only fibroblasts in the vicinity of the tumour cells would respond to it as they do in the co-culture experiments. The decrease in collagen protein, observed in the conditioned medium experiments, may have been caused partly by collagenases present in the conditioned medium after co-culture with the fibroblasts (data not shown). The decrease in collagen mRNA, however, probably involves a more complicated mechanism. The most likely explanation is that the tumour cells require either direct cell–cell contact with the fibroblasts, or need to be in very close proximity to the fibroblasts in order to bring about down-regulation of collagen mRNA. Direct cellular contact would require that the tumour cells express a cell surface molecule, which would bind a receptor on the fibroblasts leading to down-regulation of collagen gene transcription via the signal transduction pathway.

Alternatively, the tumour cells may secrete a factor, such as a cytokine, at extremely low levels such that close proximity to the target cell is required for binding to occur. The cytokines interleukin (IL)-1β, tumour necrosis factor α (TNF-α) and transforming growth factor β (TGF-β) exert their effects on type I collagen synthesis in cultured Ito cells (lipocytes) via different mechanisms. IL-1β acts at a post-transcriptional level to inhibit collagen synthesis, TNF-α inhibits the transcription rate of the proα1(I) collagen gene and TGF-β increases proα1(I) collagen gene expression by increased transcription (Armendariz-Borunda et al, 1992). These are therefore possible mechanisms, at least in the case of IL-1β and TNF-α, by which collagen gene expression could be down-regulated in the fibroblasts.

There are two general conclusions that can be drawn from this study. First, that stromal fibroblasts, not the tumour cells, are responsible for the overproduction of collagen in stage I breast tumour tissue. Secondly, that tumour cells are able to induce down-regulation of collagen mRNA production in fibroblasts in advanced stage breast tumours by a mechanism yet to be elucidated. The results suggest a novel way in which tumour cells can modulate the extracellular matrix in order to facilitate invasion and subsequent metastasis. We postulate that a physical interaction exists between tumour cells and fibroblasts which is essential for the observed down-regulation of collagen mRNA, and which, either directly or indirectly, results in decreased ECM production and ultimately disease progression.

### ACKNOWLEDGEMENTS

We thank Professor Tiltman and Dr Pauline Close (Department of Anatomical Pathology) for staging of the breast tissue; Sumayah

 *© 1999 Cancer Research Campaign*

Cornelius and Helen Ilsley (Department of Anatomical Pathology) for preparation of the tissue sections and Dr Malcolm Collins for helpful critiques of the manuscript. This work was supported by grants from the South African Medical Research Council, The Cancer Association of South Africa and The University of Cape Town.

### REFERENCES

Al-Adanani MS, Kirrane JA and McGee JOD (1975) Inappropriate production of collagen and prolyl hydroxyproline by human breast carcinoma cells in vivo. *Br J Cancer* **31**: 653–660

Armendariz-Borunda J, Katayama K and Seyer JM (1992) Transcriptional mechanisms of type I collagen gene expression are differentially regulated by interleukin 1β, tumor necrosis factor α1 and transforming growth factor β in Ito cells. *J Biol Chem* **267**: 14316–14321

Barsky SH, Rao CN, Grotendorst GR and Liotta LA (1982) Increased content of type V collagen in desmoplasia of human breast carcinoma. *Am J Pathol* **108**: 276–283

Barsky SH and Gopalakrishna R (1987) Increased invasion and spontaneous metastasis of BL6 melanoma with inhibition of the desmoplastic response. *Cancer Res* **47**: 1663–1667

Basset P, Bellocq JP, Wolf C, Stoll I, Hutin P, Limacher JM, Podhajcer OL, Chenard MP, Rio MC and Chambon P (1990) A novel metalloproteinase gene specifically expressed in stromal cells of breast carcinomas. *Nature* **348**: 699–704

Biswas C (1982) Tumor cell stimulation of collagenase production by fibroblasts. *Biochem Biophys Res Comm* **109**: 1026–1034

Biswas C (1984) Collagenase stimulation in co-culture of human fibroblasts and human tumour cells. *Cancer Lett* **24**: 201–207

Chomczynski P and Sacchi N (1987) Single-step method of RNA isolation by acid guanidinium thiocyanate-phenol-chloroform extraction. *Anal Biochem* **162**: 156–159

Chu ML, Myers JC, Bernard MP, Ding JF and Ramirez F (1982) Cloning and characterization of five overlapping cDNAs specific for the human pro alpha 1(I) collagen chain. *Nucleic Acids Res* **10**: 5925–5934

Harris JR, Lippman ME, Veronisi U and Willet W (1992) Breast cancer. *N Engl J Med* **327**: 473–480

Hewitt RE, Powe DG, Carter GI and Turner DR (1993) Desmoplasia and its relevance to colorectal tumour invasion. *Int J Cancer* **53**: 62–69

Hoefakker S, Boersma WJA and Claassen E (1995) Detection of human cytokines in situ using antibody and probe based methods. *J Immunol Methods* **185**: 149–175

Kauppila S, Stenback F, Risteli J, Jukkola A and Risteli L (1998) Aberrant type I and type III collagen gene expression in human breast cancer in vivo. *J Pathol* **186**: 262–268

Liotta LA, Rao CN and Barsky SH (1983) Tumour invasion and the extracellular matrix. *Lab Invest* **49**: 636–649

Myers JC, Chu ML, Faro SH, Clark WJ, Prockop DJ and Ramirez F (1981) Cloning a cDNA for the pro alpha 2 chain of human type I collagen. *Proc Natl Acad Sci USA* 78: 3516–3520

Nakanishi H, Oguri K, Takenaga K, Hosoda S and Okayama M (1994) Differential fibrotic stromal responses of host tissue to low- and high-metastatic cloned Lewis lung carcinoma cells. *Lab Invest* **70**: 324–332

Niitsu Y, Ito N, Kohda K, Owada M, Morita K, Sato S, Watanabe N, Kohgo Y and Urushizaki I (1988) Immunohistochemical identification of type I procollagen in tumour cells of scirrhous adenocarcinoma of the stomach. *Br J Cancer* **57**: 79–82

Noel A, Munaut C, Boulvain A, Calberg-Bacq CM, Lambert CA, Nusgeus B, Lapiere CM and Foidart JM (1992) Modulation of collagen and fibronectin synthesis in fibroblasts in normal and malignant cells. *J Cell Biochem* **48**: 150–161

Ohtani H, Kuroiwa A, Obinata M, Ooshima A and Nagura H (1992) Identification of type I collagen-producing cells in human gastrointestinal carcinomas by non-radioactive in situ hybridisation and immunoelectron microscopy. *J Histochem Cytochem* **40**: 1139–1146

Pauli BU, Schwartz DE, Thonar EJ and Knettner KE (1983) Tumor invasion and host extracellular matrix. *Cancer Metastas Rev* **2**: 129–152

Peterkofsky B and Diegelmann R (1974) Use of a mixture of proteinase-free collagenases for the specific assay of radioactive collagen in the presence of other proteins. *Biochemistry* **10**: 988–993

Pucci-Minafra I, Minafra S, Sciarrino S, Tomasino RM and Tinervia RJ (1986) Collagen changes in the ductal infiltrating (schirrous) carcinoma of the human breast. A possible role played by type I trimer collagen on the invasive growth. *Submicrosc Cytol* **18**: 795–805

Roesel RA, Cutroneo KR, Scott DF and Howard EF (1978) Collagen synthesis by cloned mouse mammary tumour cells. *Cancer Res* **38**: 3269–3275

Sakakibara K, Suzuki T, Motoyama T, Watanabe H and Nagai Y (1982) Biosynthesis of an interstitial type of collagen by cloned human gastric carcinoma cells. *Cancer Res* **42**: 2019–2027

Smith DR, Kunkel SL, Burdick MD, Wilke CA, Orringer MB, Whyte RI and Strieter RM (1994) Production of interleukin-10 by human bronchogenic carcinoma. *Am J Pathol* **145**: 18–25

van der Hooff A (1988) Stromal involvement in malignant growth. *Adv Cancer Res* **50**: 159–196

*© 1999 Cancer Research Campaign*
